# The immediate effect of cervical rotation-traction manipulation on cervical paravertebral soft tissue: a study using soft tissue tension cloud chart technology

**DOI:** 10.1186/s12891-024-07277-5

**Published:** 2024-02-29

**Authors:** Xu Wang, Zikai Jin, Tianxiao Feng, Shengjie Fang, Chuanrui Sun, Xiaokuan Qin, Kai Sun, Long Liang, Guangwei Liu, Liguo Zhu, Xu Wei

**Affiliations:** 1 Wangjing Hospital, China Academy of Chinese Medical Sciences, Beijing, China; 2 Beijing Key Laboratory of Traditional Chinese Orthopedics and Traumatology, Beijing, People’s Republic of China; 3 Beijing University of Chinese Medicine, Beijing, People’s Republic of China; 4 Anhui Provincial Hospital of Chinese Medicine, Hefei, Anhui People’s Republic of China; 5 Liaocheng Hospital of Chinese Medicine, Liaocheng, Shandong People’s Republic of China

**Keywords:** Cervical spondylotic radiculopathy, Cervical paravertebral soft tissue, Soft tissue tension cloud chart, Cervical rotation-traction manipulation

## Abstract

**Background:**

To evaluate the reliability of the Soft Tissue Tension Cloud Chart (STTCC) technology, an original method combining multi-point Cervical Paravertebral Soft Tissue Test (CPSTT) with MATLAB software, we conducted a preliminary analysis on the immediate effects of Orthopaedic Manual Therapy (OMT) on cervical paravertebral soft tissue.

**Methods:**

30 patients with Cervical Spondylotic Radiculopathy (CSR) were included in this study. We analyzed the differences in CPSTT before and after treatment with Cervical Rotation-Traction Manipulation (CRTM), a representative OMT technique in Traditional Chinese Medicine, using the STTCC technology.

**Results:**

The STTCC results demonstrated that post-treatment CPSTT levels in CSR patients were significantly lower than pre-treatment levels after application of CRTM, with a statistically significant difference (*P* < 0.001). Additionally, pre-treatment CPSTT levels on the symptomatic side (with radicular pain or numbness) were higher across the C5 to C7 vertebrae compared to the asymptomatic side (without symptoms) (*P* < 0.001). However, this difference disappeared after CRTM treatment (*P* = 0.231).

**Conclusions:**

The STTCC technology represents a reliable method for analyzing the immediate effects of OMT. CSR patients display uneven distribution of CPSTT characterized by higher tension on the symptomatic side. CRTM not only reduces overall cervical soft tissue tension in CSR patients, but can also balance the asymmetrical tension between the symptomatic and asymptomatic sides.

**Trial registration:**

This study was approved by the Chinese Clinical Trials Registry (Website: .https://www.chictr.org.cn.) on 20/04/2021 and the Registration Number is ChiCTR2100045648.

## Introduction

Neck pain is a common reason for general practitioner visits and one of major contributor to disability, as measured by Years Lived with Disability (YLDs) [1]. An epidemiological study [2] estimated over 288.7 million cases of neck pain and 28.6 million related disability cases worldwide in 2017. Cervical Spondylotic Radiculopathy (CSR), one of the primary causes of neck pain, is characterized by pain in one or both upper extremities accompanied by neck pain, secondary to neural compression or irritation in the cervical spin e[3, 4]. Due to its safety and cost-effectiveness [5–7], conservative therapy is the most common initial treatment approach for CSR and the Orthopaedic Manual Therapy (OMT) represents a key conservative treatment method. Distinct from modern medicine, Traditional Chinese Medicine (TCM) has developed a unique theoretical framework derived from extensive clinical experience summarizatio n[8] and OMT in TCM is an important part of it. Currently, as a safe and effective physiotherap y[9, 10], OMT in TCM has been widely used, and it has been included in many clinical practice guidelines for CS R[11–13]. However, analyzing treatment effects through modern technology remains an important challenge limiting the popularization of these approaches.

Recent advances in spinal biomechanic s[14, 15], neuroimagin g[16, 17], and electrophysiolog y[18, 19] have facilitated therapeutic mechanism research on OMT. However, the impact of OMT on cervical paravertebral soft tissues has been relatively underexplored due to a lack of reliable assessment indicators. Paravertebral soft tissue play a significant role in the pathogenesis and treatment of CSR [20]. In particular, increased Cervical Paravertebral Soft Tissue Tension (CPSTT) associates with neck pain symptoms [21], while physical modalities demonstrate advantages in managing this type of pain [22]. Developing objective measures and relevant instrumentation to evaluate OMT effects on paravertebral soft tissues represents an important research need.

**Fig. 1:**
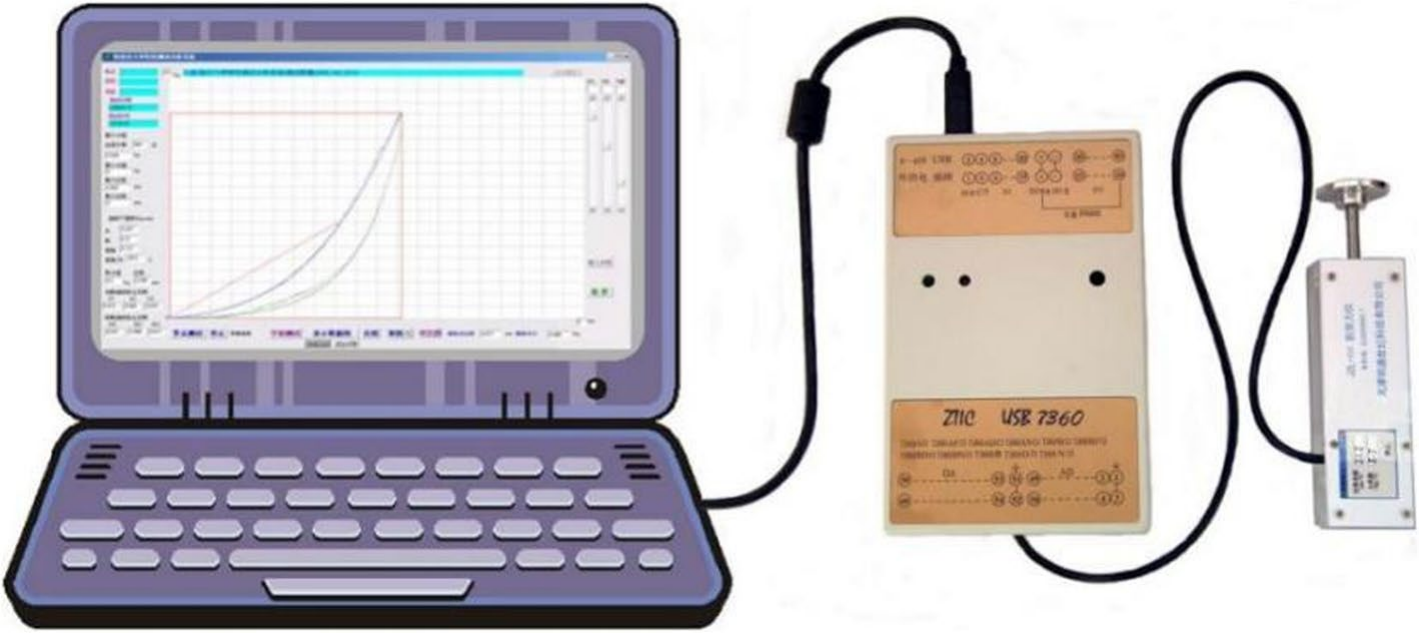
Soft tissue tension analysis system

**Fig. 2:**
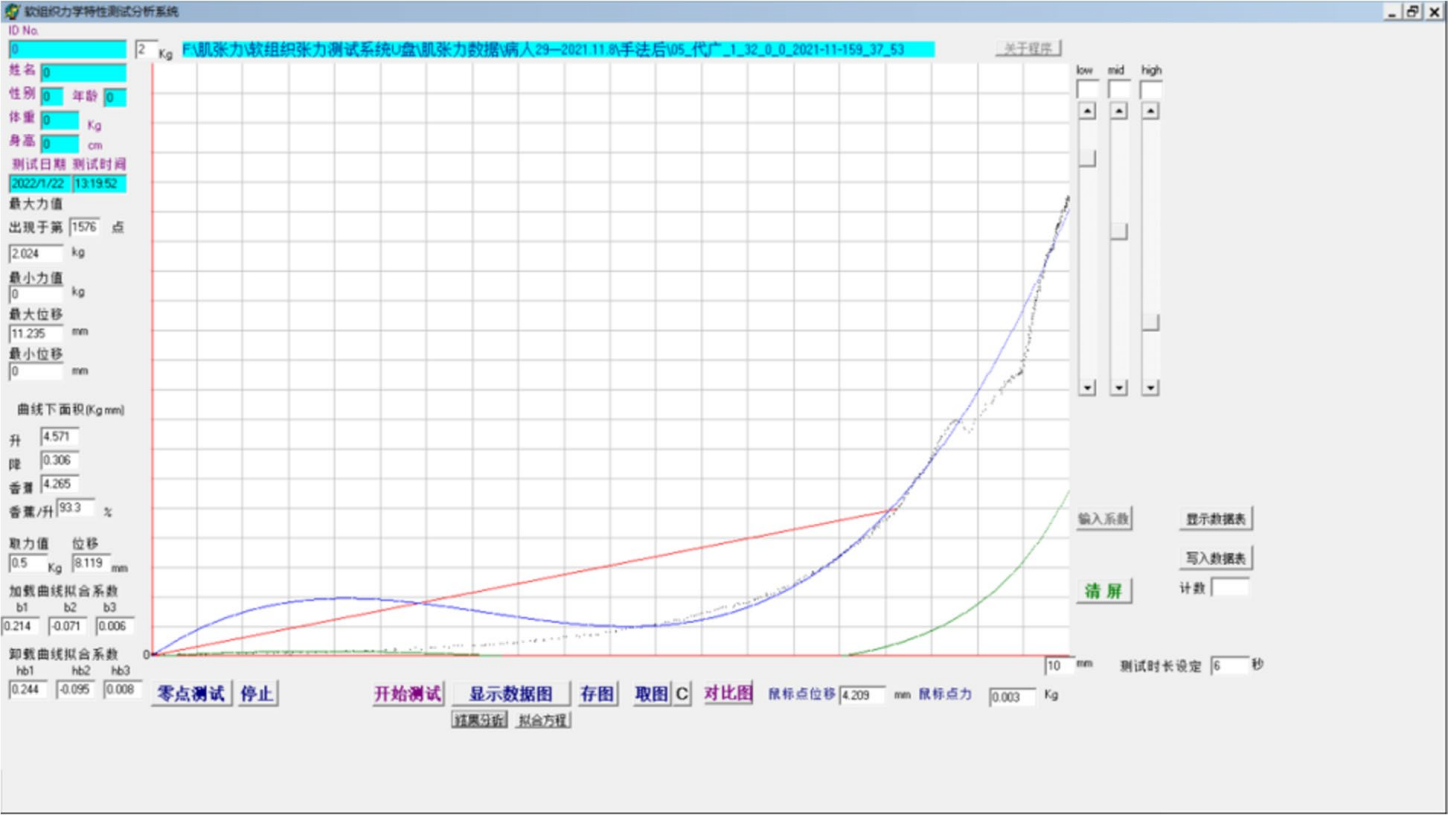
Supporting software soft tissue tension analysis system

To address this need, the Soft Tissue Tension Analysis System (STTAS, Fig. 1 and Fig. 2) was jointly developed by the Institute of Orthopedics and Traumatology and Tianjin Mingtongshiji Technology Company. This system comprises supporting software, an adapter, and a soft tissue tension probe that generates function curves depicting tissue tension at varying compression forces. The reliability of this equipment has been verifie d[23, 24]. Building on this foundation and integrating MATLAB software with multi-point CPSTT testing, we innovated the Soft Tissue Tension Cloud Chart (STTCC) technology for comprehensive and intuitive soft tissue tension assessments. Leveraging this novel methodology, we selected 30 CSR patients to analyze the immediate impacts of OMT on cervical paravertebral soft tissues, exploring an innovative technique for evaluating real-time therapy effects on paravertebral tissues.

## Materials and methods

This study was registered with the Chinese Clinical Trial Registry (Website: https://www.chictr.org.cn, Registration Number: ChiCTR2100045648) and the Ethics approval and consent to participate (Approval Number: WJEC-KTK2020–012-P002). All patients have signed informed consent. All the procedures were performed in accordance with the Declaration of Helsinki.

### Participants

From May 2021 to November 2021, 30 CSR patients were recruited for participation at Wangjing Hospital and the Orthopaedic institute of China Academy of Chinese Medical Sciences. The same physician performed CRTM treatment on all patients.

### Sample size

As an exploratory study, prior research on intervention testing typically utilizes sample sizes of 20–25 per grou p[25]. Accounting for potential subject refusal of second soft tissue tension assessments, 30 participants were enrolled.

### Inclusion criteria

Patients met the Chinese Association of Rehabilitation Medicine’s diagnostic criteria for CS R[26], with typical symptoms of neck pain and upper extremity radicular pain or numbness. Additional inclusion criteria were age 18–65 years and imaging findings of “cervical degeneration” and “foraminal stenosis”. Two chief physicians confirmed all diagnoses.

### Exclusion criteria

Patients with spinal tuberculosis, osteomyelitis, nervous system tumors, severe chronic diseases such as serious cardiovascular or cerebrovascular disease, blood diseases, liver and kidney dysfunction, cervical spondylosis myelopathy, severe osteoporosis, spinal stenosis, arm muscle strength ≤ grade 3, skin diseases or lesions at the treatment site, and a history of cervical surgery operations would be excluded. Female patients during pregnancy, lactation, and menstruation would also be excluded.

### Informed consent

Eligible patients received explanations of study purpose, methods, interventions, and assessments. Patients provided written informed consent and medical history self-reports prior to enrollment.

### Interventions

Patients underwent Cervical Rotation-Traction Manipulation (CRTM), representing OMT techniques from TCM. Standard CRTM protocols have been established with proven safety and efficacy across clinical trial s[27, 28].

**Fig. 3:**
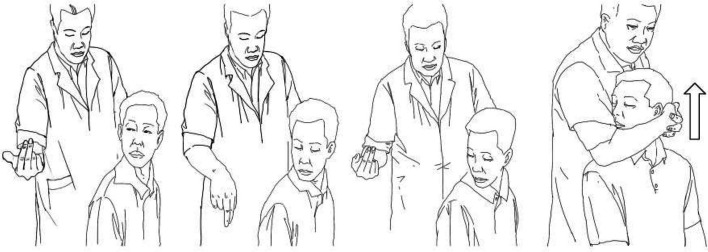
Operation process of CRTM

CRTM entails the following steps (Fig. 3): 1) 10–15 minute massage, 2) head rotation to physiological limit with slight lowering, 3) operator head embrace and pre-traction force application, and 4) operator gives a short lifting force, we can hear some snaps form the cervical joints when this manipulation is successful. The same physician performed all CRTM procedures.

### The CPSTT tests

**Fig. 4:**
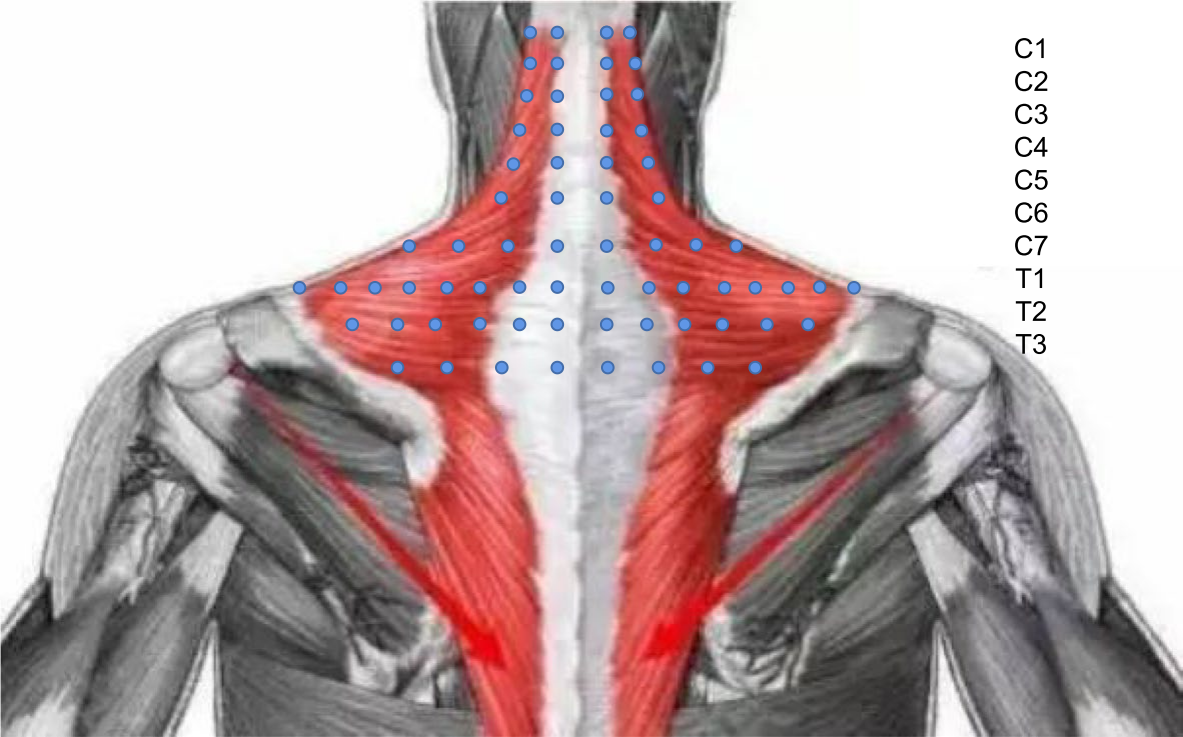
Detection points

**Fig. 5:**
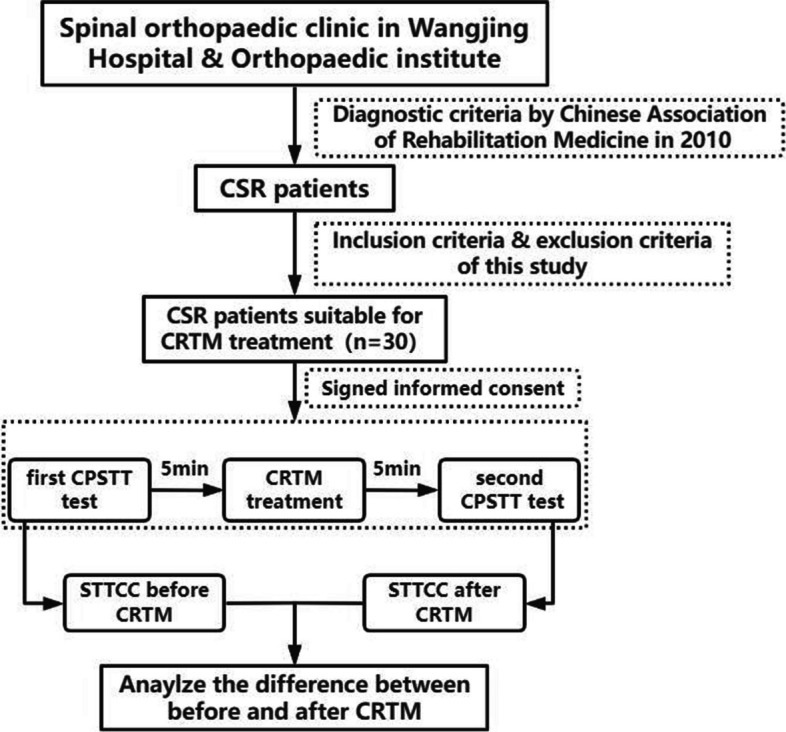
The process of soft tissue tension test

**Fig. 6:**
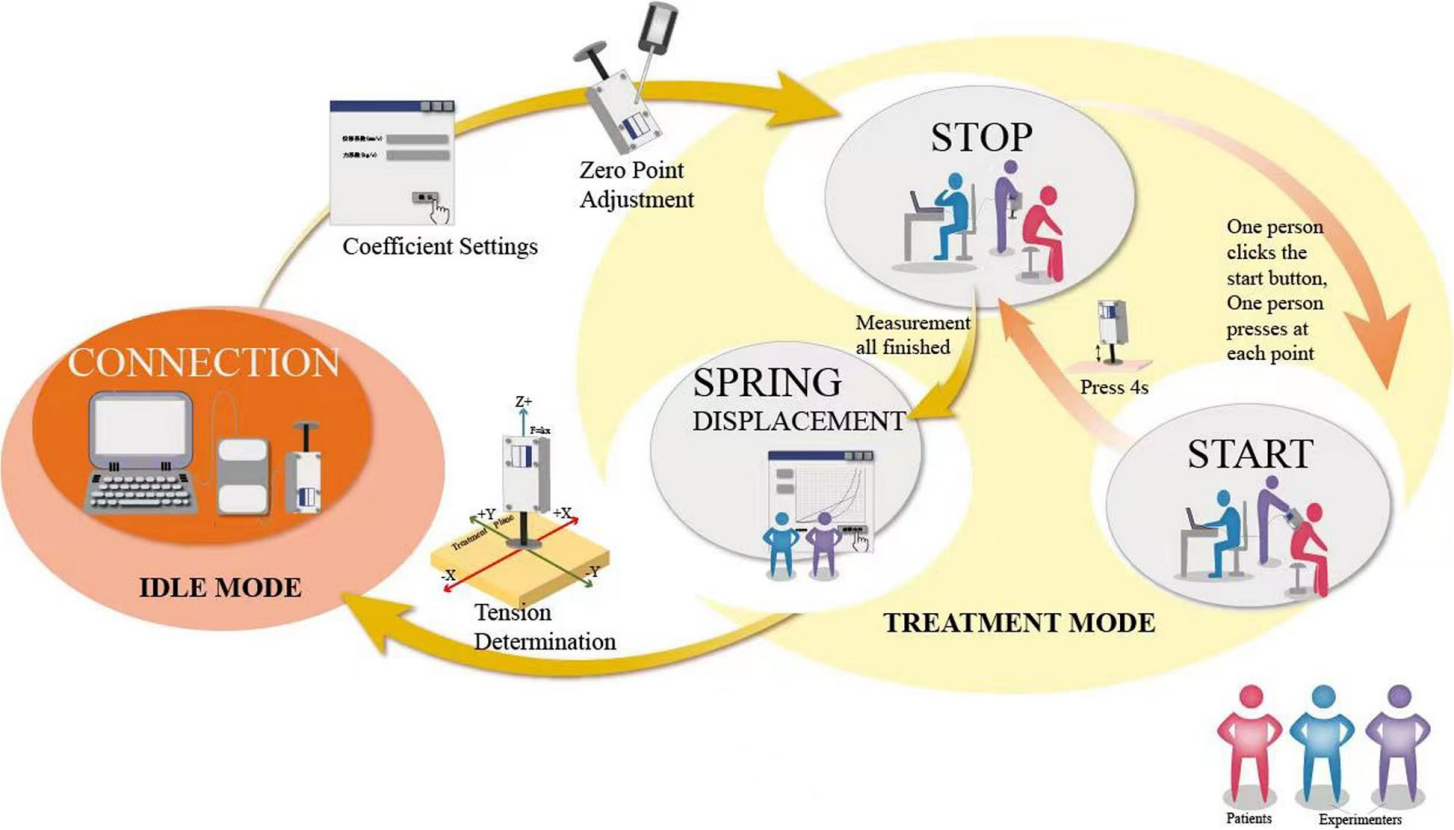
Test process diagram

Pre-treatment CPSTT assessments occurred within 5 minutes prior to massage, while post-treatment assessments took place within 5 minutes after CRTM. A total of 68 detection points (Fig. 4) are used to record the CPSTT in every test. Two operators and the patient participated in each assessment. One operator uses the probe, and the other records the data on the software (the same operator performed all probe measurements). During compression, the probe remained perpendicular to the skin surface. One software operator initiated recording, while the other operator applied and maintained constant pressure before slowly withdrawing the probe. Measurement duration lasted 4 seconds at each point (Figs. 5 and 6). Soft tissue tension calculations utilized spring displacements under 500 g compression across all detection points.

### Generating of STTCC

**Fig. 7:**
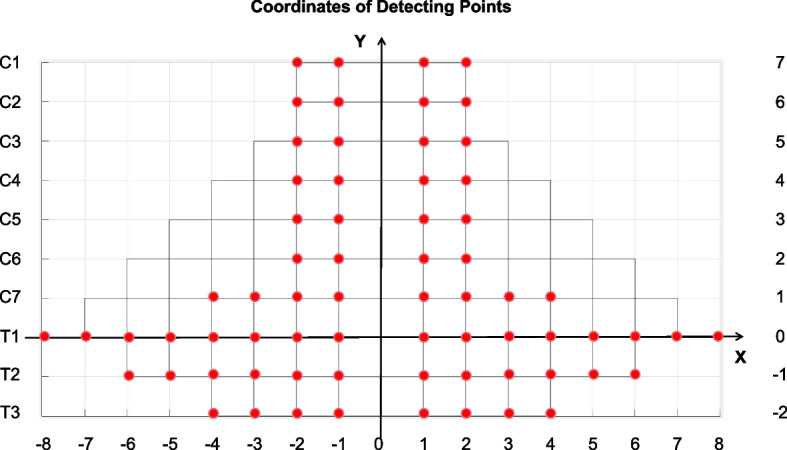
Abscissa and ordinate of detection points

The Cervical Soft Tissue Tension Cloud Charts of CPSTT are drawn by MATLAB R2015a software (MathWorks Company, U.S.A.). The first thoracic spinous process serves as the origin point of the space-rectangular coordinate system. The settings for the abscissa and ordinate are shown in Fig. 7 and the vertical is set as the probe displacements of each detection point.

### Statistical analysis and accuracy assessment

The Kolmogorov-Smirnov test (K-S test) is used to test continuous variables for a normal distribution and is presented as the median with an interquartile range. Continuous detection indices are analyzed using the ***t***-test or the Mann-Whitney test (M-W test). ***P*** < 0.05 indicates that the differences between before and after CRTM is statistically significant.

All statistical analysis is carried out with SPSS Statistics 22.0 software (International Business Machines Corporation, CA, U.S.A.). Figures are created in GraphPad Prism 8.0 (GraphPad Software, CA, U.S.A.).

## Results

### Characteristics of participants

30 CSR patients were selected for this study, including 11 males and 19 females. The mean age of these 30 patients is (49.79 ± 11.54) years old, and the median course of disease is 4.5 weeks. 8 patients experience radicular pain or numbness in their left arms, while the remaining 22 experience these symptoms in their right arms. Among them, there are 11 patients with arm radicular pain and another 19 patients with arm numbness (Table 1).

**Table 1:** Information of 30 CSR patients

No.	Gender	Age	Symptomatic side	Symptom	Course of disease	No.	Gender	Age	Symptomatic side	symptom	course of disease
1	female	46	left	radicular pain	3	16	female	59	right	numbness	18
2	female	41	right	numbness	3	17	female	38	right	radicular pain	7
3	female	48	right	numbness	8	18	male	31	right	numbness	3
4	female	63	left	radicular pain	5	19	female	48	right	numbness	4
5	male	60	right	numbness	9	20	female	33	right	radicular pain	8
6	male	56	left	radicular pain	2	21	female	41	left	numbness	5
7	female	25	right	numbness	2	22	male	55	right	numbness	4
8	female	52	right	numbness	4	23	female	63	right	numbness	8
9	male	48	right	numbness	3	24	female	58	right	numbness	10
10	female	64	right	numbness	8	25	female	60	left	radicular pain	3
11	male	64	right	numbness	14	26	female	53	right	radicular pain	5
12	female	51	right	radicular pain	11	27	male	63	right	numbness	4
13	male	33	right	radicular pain	4	28	male	53	right	radicular pain	2
14	female	62	left	numbness	8	29	male	32	left	radicular pain	2
15	female	52	left	numbness	6	30	male	44	right	numbness	2

Symptomatic side: side with arm radiation pain or numbness

Course of disease: weeks

### STTCCs before and after CRTM

**Fig. 8:**
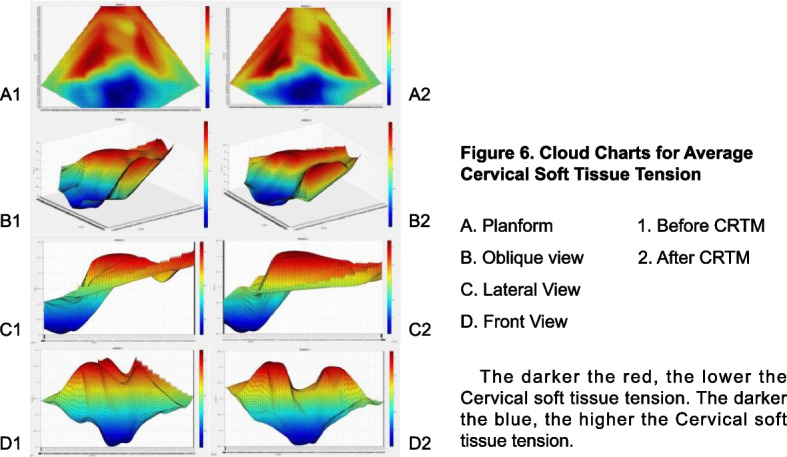
Cloud charts for average CPSTT

We average the CPSTT data of 30 patients at each point before and after CRTM respectively, and the mean data of each point is taken as the vertical coordinate to generate a STTCC (Fig. 8). The spring displacement in the red area is larger than the blue region. Hence, the red areas have lower tension and the blue areas have higher tension. According to the STTCC, it can be seen that the red area mainly includes C1-C6 level and the outward part of C7 level, while the blue area mainly includes T1-T3 level and the interior part of C7 level, which show the average tension of cervical soft tissue is lower than thoracic. Because the stiffness of the muscle belly is lower than that of the muscle tendon, two peaks can be seen on both sides of the C1-C6 level (Fig. 8, D1 and D2). The peaks before CRTM are short and sharp, and they become wider and higher after CRTM. Furthermore, when comparing before and after CRTM (Fig. 8, A1 and A2), it is discovered that soft tissue tension at the C3-C7 level, as well as the outward T1-T2 level, is significantly lower after treatment. All these phenomena show that the CPSTT after CRTM is lower than before.

### Statistical analysis of CPSTT

**Fig. 9:**
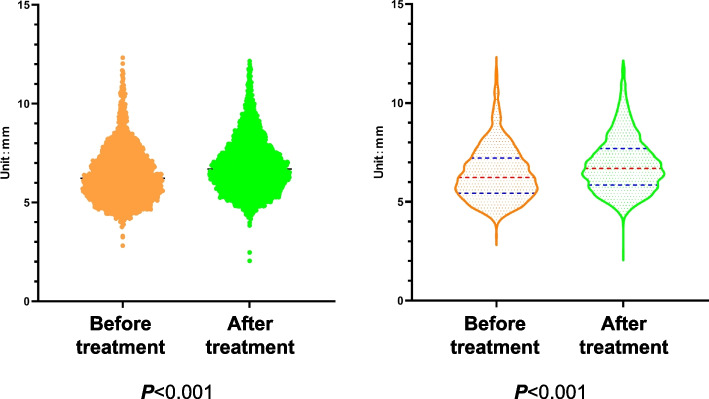
The CPSTT before and after treatment

Then, we performed statistical analyses for CPSTT at each detection point prior to and after CRTM. Firstly, all the significance values of the K-S test are lower than 0.001 (***P*** < 0.001), indicating that these data don’t conform to the normal distribution. Therefore, the M-T test is used to analyze these data. According to statistics, the median of probe data before CRTM is 6.226 mm, and the quartiles are **q**_**25**_ = 5.428 mm and **q**_**75**_ = 7.217 mm, respectively. The median of the data after CRTM is 6.695 mm, and the quartiles are **q**_**25**_ = 5.854 mm and **q**_**75**_ = 7.690 mm. The M-T test shows statistically significant differences between before and after CRTM (***P*** < 0.001), which suggests that CRTM can reduce the CPSTT of CSR patients (Fig. 9 and Table 2).

**Table 2:** M-W test for before and after CRTM

All detecting points (*n* = 4080)	Before CRTM (*n* = 2040)	After CRTM (*n* = 2040)	Z	P
6.469 (5.626, 7.456)	6.226 (5.428, 7.217)	6.695 (5.854, 7.690)	11.388	<0.001^**^

Unit: mm

^*^0.001 ≤ *P* < 0.05, ^**^P < 0.001

### CPSTT characteristics of CSR patients

Meanwhile, to explore the relationship between CPSTT and radicular pain or numbness, we analyze the tension data of the symptomatic side (with radicular pain or numbness) and the asymptomatic side (without symptoms) before and after CRTM. According to the distribution area of the brachial plexu s[29], we mainly analyze the CPSTT at the C5-C7 level.

**Fig. 10:**
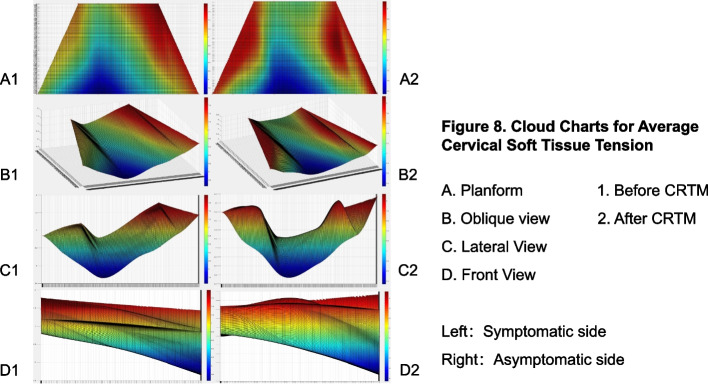
Cloud charts for average CPSTT

**Fig. 11:**
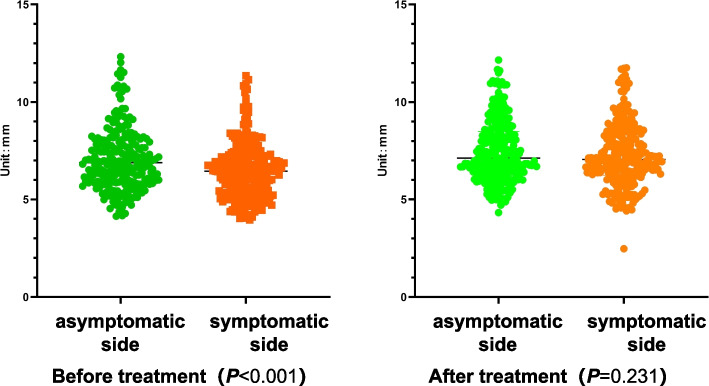
The compare between asymptomatic side and symptomatic side

The STTCCs are drawn in the same way. To make the distinction between the symptomatic and asymptomatic sides more obvious, all data from the asymptomatic side was placed on the left side of cloud charts, while data from the symptomatic side was placed on the right. The results are shown in Fig. 10. We can see the differences: the CPSTT of the symptomatic side is obviously higher than that of the asymptomatic side before treatment, and the differences decrease after treatment. Statistically, the K-S test suggests that the CPSTT at the C5-C7 level doesn’t conform to a normal distribution (***P*** < 0.001). According to the result of the M-T test, the median of the symptomatic side data at the C5-C7 level before CRTM is 6.450 mm, and the quartiles are 5.360 mm (**q**_**25**_) and 7.230 mm (**q**_**75**_). The asymptomatic side data are 6.890 mm, 5.930 mm (**q**_**25**_), and 8.028 mm (**q**_**75**_). The M-T test shows a statistically significant difference between before and after CRTM (***P*** < 0.001). After CRTM, the symptomatic side median is 7.054 mm, and the quartiles are 6.245 mm (**q**_**25**_) and 8.316 mm (**q**_**75**_). The asymptomatic side data are 7.121 mm, 6.304 mm (**q**_**25**_), and 8.483 mm (**q**_**75**_). The difference isn’t statistically significant (***P*** = 0.231). In addition, both the differences before and after CRTM on the symptomatic side and the asymptomatic side are statistically significant (***P*** < 0.001, ***P*** = 0.008) (Table 3 and Fig. 11). These results show a correlation between soft tissue tension at C5-C7 levels and symptoms of radicular pain or numbness. After treatment, both the CPSTT of the symptomatic side and the asymptomatic side have significantly improved, while the difference between the symptomatic side and the asymptomatic side has no statistical significance, which suggests that CRTM can reduce and balance the CPSTT of CSR patients.

**Table 3:** M-W test for symptomatic side and asymptomatic side

	symptomatic side(*n* = 240)	asymptomatic side(n = 240)	Z	P
Before CRTM	6.450 (5.360，7.230)	6.890 (5.930，8.028)	−4.130	<0.001^**^
After CRTM	7.054 (6.245, 8.316)	7.121 (6.304, 8.483)	−1.197	0.231
Z	5.405	2.645		
P	<0.001^**^	0.008^*^		

Unit: mm

*0.001≤ *P*<0.05, **P<0.001

## Discussion

Neck pain is one of the top five chronic pain conditions in terms of prevalence and years lost to disabilit y[30], causing a huge economic burden every yea r[31], and CSR is a common disease that causes it. As an economical and applicable treatmen t[32, 33], physical therapy, such as OMT or acupuncture, is widely recommended for neck pain patients, especially in low- and middle-income communitie s[34]. The immediate effect of OMT on cervical muscles or soft tissue is one of the main fields of study in understanding the mechanism of OMT. In this study, we evaluated the reliability of the original STTCC technology and preliminarily identified the immediate effect of CRTM on the overall reduction and balanced distribution of CPSTT in patients with CSR.

### Application prospect of STTCC technology

In fact, there are many evaluating tools available to describe soft tissue tension or muscle tension with different theories, such as Myotonometr y[35, 36], MyotonPR O[37, 38], Shear Wave Elastograph y[39, 40], and the STTAS in this study. All these tools can be used to evaluate and analyze the stiffness of soft tissue. However, it is a problem figuring out how to describe the data generated by these technologies in an intuitive and comprehensive way. Currently, many researchers evaluate the stiffness of one muscle using the single-point muscle tension tes t[41–43], which cannot fully reflect the overall condition of the muscle, especially when it is used to evaluate the effect of OMT. Hence, we combined MATLAB software with the multi-point CPSTT test and created the STTCC technology (Fig. 8 and Fig. 10). Compared with simple data analysis, this method can reflect the change of soft tissue tension and the distribution of soft tissue tension between each detection point more directly. Though Shear Wave Elastography technology can achieve a similar effect, it needs a professional ultrasound doctor to coordinate and analyze. Meanwhile, STTCC technology can be used to describe the data formed by myotonometry and MyotonPRO, which is a supplement for the traditional methods of muscle stiffness testing. In addition, as long as the detection points are selected properly and the coordinates are set properly, this method can be used to describe muscle stiffness or soft tissue tension in many places, such as the knee, back, arm, or leg.

### CPSTT and neck pain

Cervical musculoskeletal tissues, particularly the muscles, critically maintain spinal stabilit y[44]. Biomechanical research shows complex interplay between passive and active stabilizing structure s[45]. Effective control over muscular force and stiffness helps preserve kinematic stabilit y[46]. It has been found that muscle force and stiffness were proportional to the dynamic stability of the spin e[47], which confirms the protective effect of muscles on spine stability. However, muscular fatigue and strain on surrounding soft tissues can provoke mechanical neck pai n[4], representing the initial phase of CSR progression and degeneratio n[48]. Certain lifestyle factors may exacerbate these processes, enhancing disease chronicit y[49]. Additionally, injuries or structural damage can hasten cervical degeneration. Patients sustaining cervical soft tissue damage develop significantly more frequent long-term degenerative changes compared to the general populatio n[50]. After lower trapezius muscle strengthening exercises, patients with neck pain demonstrate significant improvements in muscle morphology and function corresponding to reduced symptom s[51]. Multiple studies confirm associations between heightened cervical muscle stiffness and neck pai n[52–55]. These collective findings underscore critical cervical soft tissue involvement in neck pain and substantiate the importance of targeted muscle interventions for CSR.

### CRTM has immediate effects of reducing and balancing CPSTT

Our results demonstrate CRTM effectively decreases CPSTT in CSR patients (***P*** < 0.001), corroborating prior investigations on OMT-induced muscle relaxation and pain relie f[56, 57]. However, one study in asymptomatic volunteers found no impact of cervical manipulation on muscle tone, elasticity, or stiffness despite modulating pressure pain threshold s[58]. The discrepancy likely reflects differences between healthy participants versus CSR patients with baseline muscle dysfunction. Hence, we believe our conclusions on CRTM-mediated CPSTT reductions in the target patient population remain valid.

Meanwhile, interestingly, we also find a significant difference in soft tissue tension between CSR patients symptomatic side (with radicular pain or numbness) and asymptomatic side (C5 to C7, ***P*** < 0.001). This difference, as well as the standard deviation of CPSTT, disappeared after the CRTM interventions, implying that CRTM can balance soft tissue tension on the symptomatic and asymptomatic sides. We find a few studies [59] with similar results to this conclusion. On the contrary, some studie s[60, 61] discovered that the cervical muscle thickness and cross-sectional area of the symptomatic side were lower in chronic CSR patients than the asymptomatic side. But the relationship between soft tissue tension and muscle thickness has not been established. Therefore, further studies are needed to confirm this field.

### Study advantages and limitations

Utilizing our original STTCC analysis technology, we provide intuitive visualization of CRTM impacts on CPSTT in CSR patients. Investigating associations between CRTM and CPSTT advances the objectification of TCM technique assessments while exploring potential alleviation mechanisms. We also characterize preliminarily correlations between heightened CPSTT and radicular CSR symptoms, alongside CRTM balancing effects for CPSTT.

As an initial investigation, limitations include our modest sample size (*N* = 30) constraining result generalizability. We lacked age or symptom duration stratification, spanning a wide age range that could obscure tension profiles unique to certain demographics. Moreover, OMT is usually criticized that the effect is not sustainable, but our work haven’t yet used this technology to verify the long-term intervention effect of OMT at this stage. Nevertheless, our central novel contributions entail developing an innovative methodology for analyzing and visually representing CRTM consequences on cervical soft tissues while elucidating initial evidence for CRTM-mediated CPSTT reduction and balancing in the target population. These foundations pave the way for future mechanistic research on TCM techniques.

## Conclusion

Leveraging our novel STTCC technology, current findings indicate CRTM decreases and balances CPSTT in CSR patients, potentially alleviating neck pain. Meanwhile, the STTCC represents a reliable analytical approach for quantifying and visualizing muscle stiffness.

## Abbreviations

CSR: Cervical Spondylotic Radiculopathy
YLDs: Years Lived with Disability
OMT: Orthopaedic Manual Therapy
TCM: Traditional Chinese Medicine
CRTM: Cervical Rotation-Traction Manipulation
CPSTT: Cervical Paravertebral Soft Tissue Tension
STTAS: Soft Tissue Tension Analysis System
STTCC: Soft Tissue Tension Cloud Chart
K-S test: Kolmogorov-Smirnov test
M-T test: Mann-Whitney ***U***-test
